# A confirmatory factor analysis of the metabolic syndrome in adolescents: an examination of sex and racial/ethnic differences

**DOI:** 10.1186/1475-2840-11-128

**Published:** 2012-10-13

**Authors:** Matthew J Gurka, Christa L Ice, Shumei S Sun, Mark D DeBoer

**Affiliations:** 1 Department of Biostatistics, School of Public Health, West Virginia University, Morgantown, West Virginia, USA; 2 Department of Biostatistics, School of Medicine, Virginia Commonwealth University, Richmond, Virginia, USA; 3 Department of Pediatrics, School of Medicine, University of Virginia, Charlottesville, Virginia, USA

**Keywords:** Metabolic syndrome, Factor analysis, Statistical, Insulin resistance, Pediatrics, Adolescents, Epidemiology, Clinical studies, Obesity, Risk factors

## Abstract

**Objective:**

The metabolic syndrome (MetS) is a cluster of clinical indices that signals increased risk for cardiovascular disease and Type 2 diabetes. The diagnosis of MetS is typically based on cut-off points for various components, e.g. waist circumference and blood pressure. Because current MetS criteria result in racial/ethnic discrepancies, our goal was to use confirmatory factor analysis to delineate differential contributions to MetS by sub-group.

**Research Design and Methods:**

Using 1999–2010 data from the National Health and Nutrition Examination Survey (NHANES), we performed a confirmatory factor analysis of a single MetS factor that allowed differential loadings across sex and race/ethnicity, resulting in a continuous MetS risk score that is sex and race/ethnicity-specific.

**Results:**

Loadings to the MetS score differed by racial/ethnic and gender subgroup with respect to triglycerides and HDL-cholesterol. ROC-curve analysis revealed high area-under-the-curve concordance with MetS by traditional criteria (0.96), and with elevations in MetS-associated risk markers, including high-sensitivity C-reactive protein (0.71), uric acid (0.75) and fasting insulin (0.82). Using a cut off for this score derived from ROC-curve analysis, the MetS risk score exhibited increased sensitivity for predicting elevations in ≥2 of these risk markers as compared with traditional pediatric MetS criteria.

**Conclusions:**

The equations from this sex- and race/ethnicity-specific analysis provide a clinically-accessible and interpretable continuous measure of MetS that can be used to identify children at higher risk for developing adult diseases related to MetS, who could then be targeted for intervention. These equations also provide a powerful new outcome for use in childhood obesity and MetS research.

## Background

The metabolic syndrome (MetS) is a cluster of interrelated individual factors that increase risk for future Type 2 diabetes mellitus (T2DM) and cardiovascular disease (CVD) [1,2]. These individual components of MetS include elevations in adiposity, triglycerides, blood pressure (BP) and fasting glucose, and low levels of high-density lipoprotein (HDL) particles (a surrogate for which is HDL cholesterol) [3]. While the pathophysiologic processes that drive abnormalities in these individual components are not fully understood, these underlying processes appear to be related to systemic insulin resistance [3]. In an attempt to understand the existence of MetS and the contributions of clinical measures of its components more fully, numerous researchers have used factor analysis, a model that explains the correlation among a set of variables in terms of a smaller set of unobserved “factors” [4-6]. A previous review highlighted the motivations and the pitfalls of the use of various forms of factor analysis for this purpose [4]. Namely, the majority of studies have been exploratory, have not fully evaluated the appropriateness of a linear factor model in exploring MetS, and have not taken great care in the factor analysis itself, for example including in the model(s) highly correlated variables such as systolic blood pressure (SBP) and diastolic blood pressure simultaneously.

For most sets of MetS criteria—including the Adult Treatment Panel III (ATP-III)—MetS is classified based on cut-off values for each of the individual components [3,7-9]. A given person is classified as having MetS in the presence of three or more abnormalities in these components. When applied to populations, the ATP-III MetS criteria for adults [3]—or an adolescent adaptation of these criteria [7]—predict elevations in measures of obesity-related inflammation (such as serum levels of high-sensitivity C-reactive protein, hsCRP [10]), oxidative stress (such as uric acid [11,12]) and insulin resistance (often assessed in epidemiological studies as fasting insulin [13-15]). In a prospective manner, a classification of MetS can be tracked over time [16] and predicts future T2DM in adolescents [8] and future CVD [2,17] and T2DM [18] in adults. As such, the presence of MetS in this era of pediatric obesity [19] has been proposed as a trigger for increased intervention [9,20].

Nevertheless, controversy exists over which of a variety of sets of MetS criteria to use among adolescents (Additional file 1: Table S1) [7,9,21-24], and evidence suggests that current criteria exhibit racial/ethnic and gender differences in the ability of MetS criteria to identify increased risk [25]. Non-Hispanic-black individuals have lower rates of MetS [26,27] despite having higher rates T2DM and death from CVD [28]. Currently-used criteria for MetS exhibit a lower sensitivity to detect insulin resistance [14], underlying inflammation [29] and oxidative stress (as assessed by levels of uric acid) [30] among non-Hispanic-black adolescents compared to non-Hispanic whites or Hispanics. A major reason for this is that in non-Hispanic-black adolescents triglyceride levels are lower and HDL levels are higher (i.e., more favorable) at baseline, and although these levels continue to worsen with progressive insulin resistance, they are overall less likely to exceed population cut-offs required for MetS diagnosis [27,31,32].

Because of these drawbacks of current MetS criteria, many have advocated for using a continuous scale for MetS diagnosis [33,34] or for criteria that are race/ethnicity-specific [27,31,32] Eisenmann provides an overview of multiple proposed continuous pediatric MetS scores, the majority of which use a sum of z-scores of individual MetS components [33]. While these z-scores can be calculated to account for age, sex and race/ethnicity, the method of standardizing components (e.g., waist circumference (WC), blood pressure) and summing the resulting z-scores to formulate a MetS score does not account for the strong correlations that occur across the components themselves and does not account for possible differential influences of individual components on the overall score [33]. To better account for these drawbacks, Li and Ford performed a confirmatory factor analysis on adolescents to assess the validity of one factor in explaining the covariance across the traditional MetS components; however, they did not detect hypothesized differences by sex and race/ethnicity, potentially due to being inadequately powered [5]. Others have performed such an analysis in ethnically homogeneous populations [6,35].

Our study had two primary goals. Our first goal was to perform a multi-group confirmatory factor analysis that assumed a one-factor model of the traditional MetS components while allowing for differences across sex and race/ethnicity [5]. Thus, we are testing our hypothesis that the factor structure is the same across all sex and race/ethnicity groups, but that the manner in which these MetS components correlate with this factor differs across the groups in a meaningful way. Second, if we found differences in these correlations utilizing this one-factor model, we would utilize the factor score from this model among adolescents as a continuous MetS “risk score” that was sex- and race/ethnicity specific. The ability of this risk score to detect elevations in surrogate factors related to processes underlying MetS, including hsCRP, uric acid and fasting insulin, could then be assessed on a race/ethnicity-specific basis. The data for this study were from the past twelve complete years released from NHANES (1999–2010), and thus would provide substantially more power than prior attempts to detect sex and racial/ethnic differences if they exist and would provide a subsequent reliable set of equations from which to base a continuous score. Our hypothesis was that such a race/ethnicity and sex-specific score, if determined necessary by the confirmatory factor analysis, would perform better than traditional MetS criteria at predicting surrogate factors related to MetS.

## Methods and procedures

Data were obtained from NHANES (1999–2010), a complex, multistage probability sample of the US population [7]. These annual cross-sectional surveys are conducted by the National Center for Health Statistics (NCHS) of the Centers for Disease Control (CDC), with randomly-selected subjects undergoing anthropometric and blood pressure measurements, answering questionnaires and undergoing phlebotomy. The NCHS ethics review board reviewed and approved the survey and participants gave informed consent prior to participation. Body mass index (BMI), SBP, and laboratory measures of triglycerides, HDL-C, and fasting glucose were obtained using standardized protocols and calibrated equipment [7]. For SBP, the mean of up to four readings taken on each individual was used. All blood samples used for analyses were obtained following a fast ≥8 hours prior to the blood draw.

Data from non-Hispanic-white, non-Hispanic-black, or Hispanic (Mexican-American/other Hispanic) adolescents 12–19 years old were analyzed. Children <12 years old were excluded since fasting values for triglycerides and glucose were only obtained in participants ≥12 years old. Subjects were excluded if they had known diabetes or unknown diabetes (fasting plasma glucose >125 mg/dL), as each of these limit insulin release [36]. Pregnant women were also excluded, as well as individuals taking antihyperlipidemic or anti-diabetic medications as these are all likely to alter lipid and insulin levels.

We combined all data sets from the 6 two-year cycles (1999–2010) for statistical analyses to increase our total sample size. Prevalence rates of MetS were calculated by sex and race/ethnicity, according to Ford’s pediatric adaptation of the ATP III adult criteria [7], and mean levels (95% CI’s) of the MetS components of interest (BMI z-score, SBP, HDL, triglycerides, and glucose) as well as the surrogate outcomes (hsCRP, uric acid, fasting insulin) were calculated by sex and race/ethnicity.

A series of confirmatory factor analyses (CFA) were then performed on the five identified MetS components: BMI z-score, SBP, HDL, triglycerides, and glucose. Numerous definitions of MetS have been proposed with various components that comprise it [3,7-9,21-24,37-39]. We selected these five components exclusively because of their inclusion in the most prominently used MetS criteria (Additional file 1: Table S1) and due to their clinical accessibility. We chose to use BMI-z-score instead of WC because BMI-z-scores can be standardized by age and sex using established CDC programs [40]. Unfortunately, while cut-off values exist to identify elevations in WC by age in adolescents [7,9], there is a lack of precise percentiles to permit such standardization of WC values in adolescents. In addition, WC as a clinical measure is prone to substantial measurement error [41] and is not consistently used in clinical care, while BMI z-scores are readily available and are the predominant tool recommended for adiposity assessment in clinical practice [42]. With such a factor analysis, both SBP and DBP should not be included together [4], and we chose SBP given it is more strongly associated with insulin resistance [5] and other outcomes [43]. Triglycerides were log-transformed, and all variables were standardized (mean=0, SD=1) over the entire sample. The inverse of HDL was used when standardizing, so a higher factor loading score would be similar in interpretation to the other measures in the model. The variables were not standardized within groups to allow for potential overall higher standardized scores within sex- and race/ethnic-specific groups. A one-factor model formed the basis of all of the CFA’s performed; measurement errors between the five components were assumed not to be correlated. The factor loadings were of interest, indicating the magnitude of association between each component and the underlying “MetS” factor. Factor loadings >0.3 were considered clinically meaningful. Using PROC CALIS in SAS, the parameters of the CFA’s were estimated via maximum likelihood. Two multi-group one-factor CFA’s were fit:

1) Model 1: constrained the factor loadings to be equal across the six combinations of sex and race/ethnicity;

2) Model 2: allowed the factor loadings to vary across the six groups.

Chi-square tests of the equality of the factor loadings across the six groups in Model 2 were performed. The two models were compared using various fit statistics, both overall and by group. Chi-square and Akaike’s Information Criteria (AIC) were used for model comparisons; smaller chi-square and AIC values indicated a better fit. A chi-square difference test was calculated; a significant difference between two nested models implies that the model with more paths explains the data better [44]. Other goodness of fit indices included the Root Mean Square Error of Approximation (RMSEA; >0.06 poor fit), the Standardized Root Mean Square Residual (SRMR; >0.08 poor fit), the Goodness of Fit Index (GFI; <0.90 poor fit), and the Bentler-Bonett Normed Fit Index (NFI; <0.90 poor fit) [45].

The standardized factor coefficients from the better-fitting model were used to calculate the MetS factor score on each individual. This score can be interpreted as a Z-score (mean 0, SD=1), with higher scores representing an increased risk of MetS. Receiver operating characteristic (ROC) analysis was used to assess the ability of this new score to discriminate against the traditional MetS criteria [7] as well as elevated levels of the three identified CVD/T2DM surrogates. Specifically, we were interested in the ability to predict elevated fasting insulin (>16 IU/mL, the 95^th^ percentile among normal-weight adolescents [14]), elevated hsCRP (>4.5 mg/L [29]), and elevated uric acid (approximately the 95^th^ percentile among lean individuals: 7.0 mg/dL for males, 5.5 mg/dL for females [30]). In this analysis, individuals with CRP >10.0 mg/L were excluded. In addition, the ability to predict 1 and ≥2 elevations amongst these surrogates was of interest, as this indicates the more at-risk adolescents. Overall predictive performance was measured by the area under the curve (AUC) of the ROC curve, with 0.5 and 1.0 indicating no and perfect predictive ability, respectively. We considered AUC values >0.70 to be reasonably accurate and AUC >0.90 to be very accurate. Sensitivities and specificities to predict ≥2 elevations among the three surrogates were compared between the traditional MetS classification and a definition of MetS using a cutoff identified by the ROC analysis; these statistics were done on a sex and race/ethnicity-specific basis.

Statistical significance was defined as a p-value<0.05. Statistical analysis was performed using SAS (version 9.3, Cary, NC). Descriptive statistics as well as sensitivity estimates used SAS survey procedures (SURVEYMEANS, SURVEYFREQ), which accounts for the survey design when estimating standard errors to obtain population-based estimates. The CFA itself did not account for the survey design due to the inability of standard software to perform multigroup CFA’s within subpopulations while accounting for the survey design.

## Results

Of the 4,413 participants eligible per the criteria described above, 239 (5.4%) were missing at least one of the MetS components and were not included in the analysis. The sample of participants thus consisted of 4,174 male and female non-Hispanic blacks, non-Hispanic whites, and Hispanics age 12–19 years old with complete data for MetS components (Table 1). The overall prevalence of MetS in these adolescents was 8.1% according to Ford’s pediatric adaptation of the ATP-III criteria [7]. Among US adolescent males and females, the prevalence of ATP-III-based MetS was considerably lower among non-Hispanic-blacks. Males were generally more likely to have MetS across racial/ethnic group. 

**Table 1:** NHANES 1999–2010 Characteristics: Children 12–19 Years Old with Data on all Metabolic Syndrome Components (n = 4,174)

			**Mean (95% Confidence Interval)**							
	**n**	% with Mets (95% CI)*	**BMI Z-score**	**HDL**	**Triglycerides**	**SBP**	**Glucose**	**CRP**	**Insulin**	**Uric Acid**
Overall	4,174	8.1	0.6	51.4	88.2	109.5	93.0	1.6	11.3	5.2
		(6.8, 9.4)	(0.5, 0.6)	(50.8, 51.9)	(85.6, 90.8)	(108.9, 110.1)	(92.6, 93.4)	(1.4, 1.7)	(10.9, 11.7)	(5.1, 5.2)
*By Gender and Race/Ethnicity*										
Males										
Non-Hispanic White	645	11.4	0.5	48.0	94.4	112.1	94.9	1.2	10.3	5.8
		(8.5, 14.3)	(0.4, 0.6)	(47.0, 49.0)	(89.8, 98.9)	(111.1, 113.0)	(94.3, 95.5)	(1.0, 1.4)	(9.6, 11.1)	(5.8, 5.9)
Hispanic	866	12.9	0.7	49.1	92.6	111.4	96.0	1.9	12.9	5.6
		(9.5, 16.3)	(0.6, 0.8)	(48.0, 50.2)	(88.0, 97.2)	(110.1, 112.7)	(95.2, 96.7)	(1.4, 2.3)	(11.8, 14.1)	(5.5, 5.8)
Non-Hispanic Black	697	4.9	0.6	54.9	68.9	114.6	92.8	1.4	11.1	5.3
		(3.0, 6.7)	(0.5, 0.7)	(53.5, 56.2)	(65.9, 72.0)	(113.7, 115.5)	(92.1, 93.4)	(1.1, 1.8)	(10.0, 12.2)	(5.2, 5.4)
Females										
Non-Hispanic White	566	5.0	0.5	53.3	89.1	106.1	91.4	1.7	10.5	4.7
		(3.3, 6.7)	(0.4, 0.6)	(52.2, 54.4)	(84.2, 94.0)	(105.1, 107.1)	(90.6, 92.1)	(1.3, 2.2)	(9.7, 11.3)	(4.6, 4.7)
Hispanic	831	8.2	0.7	52.2	93.9	106.0	91.9	2.1	13.7	4.4
		(5.5, 10.9)	(0.6, 0.8)	(51.2, 53.2)	(84.4, 103.5)	(105.2, 106.8)	(91.1, 92.7)	(1.7, 2.4)	(12.6, 14.7)	(4.3, 4.5)
Non-Hispanic Black	569	3.5	0.9	56.4	64.9	109.1	89.4	1.9	14.0	4.3
		(1.8, 5.3)	(0.8, 1.0)	(55.0, 57.9)	(61.9, 67.9)	(107.9, 110.2)	(88.6, 90.2)	(1.6, 2.3)	(13.1, 14.9)	(4.2, 4.4)

* Pediatric Adaptation of ATP III of Metabolic Syndrome (Ford 2007).

Comparison of overall chi-square test values between the two CFA models indicate that Model 2 provides the significantly better fit relative to the other two (*Δ*χ^2^ (50)=253.28, p<0.001); this is confirmed when comparing the Akaike’s Information Criteria (AIC) between the two overall models. The other fit statistics, presented in Table 2, indicate that while a one-factor model of the traditional MetS components is not necessarily an excellent fit, allowing the loadings to vary by sex and race/ethnicity in such a one-factor representation of MetS (Model 2) provided a better fit. When examining MetS on a sex and racial/ethnic specific basis via Model 2; a one-factor model of MetS using traditional measures fit all groups well, except for non-Hispanic-white females. Tests of the equality of the loadings across groups in Model 2 were significant for HDL, SBP and triglycerides (Table 2). Examination of the loadings in Model 2 indicate that overall glucose is not correlated with a single MetS factor for any subgroup (all loadings <0.30); we chose to keep it in the model given its prominence in established MetS criteria. SBP is more correlated with a single MetS factor for non-Hispanic-white males; HDL is less correlated with a single MetS factor for non-Hispanic-white females. Triglycerides are less correlated for females than for males, particularly for non-Hispanic-white and non-Hispanic-black adolescents.

**Table 2:** Confirmatory Factor Analysis Results*

	**Model 1**							**Model 2**						
		**Males**			**Females**				**Males**			**Females**		
**Model Fit Indices**	**Overall**	**NHW**	**NHB**	**Hisp**	**NHW**	**NHB**	**Hisp**	**Overall**	**NHW**	**NHB**	**Hisp**	**NHW**	**NHB**	**Hisp**
Chi-square (df)	492.88 (80)							239.60 (30)						
Akaike’s Information Criteria (AIC)	512.9							359.60						
Root Mean Square Error of Approximation (RMSEA)	0.086							0.100						
Standardized Root Mean Square Residual (SRMR)	0.105	0.115	0.083	0.091	0.120	0.130	0.094	0.049	0.055	0.045	0.038	0.059	0.051	0.046
Goodness of Fit Index (GFI)	0.955	0.942	0.962	0.966	0.950	0.940	0.960	0.977	0.966	0.980	0.986	0.972	0.976	0.979
Bentler-Bonett Normed Fit Index (NFI)	0.757	0.745	0.780	0.847	0.524	0.614	0.800	0.882	0.863	0.890	0.945	0.741	0.841	0.887
Factor Loadings								p-value**						
BMI z-score		0.60						0.279	0.63	0.55	0.66	0.60	0.68	0.56
SBP		0.34						0.019	0.50	0.33	0.30	0.37	0.31	0.29
HDL		0.57						0.004	0.51	0.66	0.61	0.38	0.55	0.55
Triglycerides		0.52						< 0.001	0.62	0.50	0.67	0.26	0.33	0.55
Glucose		0.21						0.250	0.23	0.27	0.22	0.11	0.18	0.20

* Model 1: Single factor, loadings constrained to be equal across all six sex-race/ethnicity groups.

Model 2: Single factor, loadings allowed to vary across all six groups.

** Chi-square test (5 df) p-value comparing factor loadings across the six groups.

NHW = Non-Hispanic White; NHB = Non-Hispanic Black; Hisp = Hispanic.

**Figure 1:**
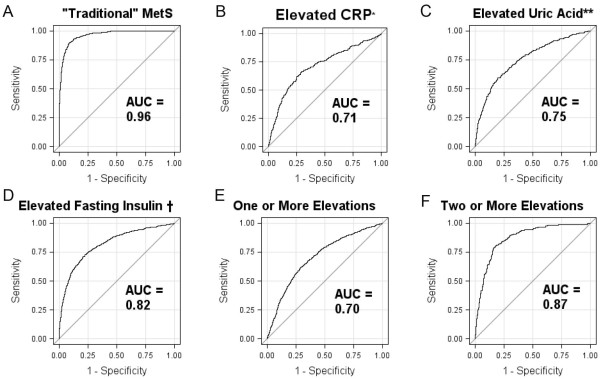
**ROC Curves for New Sex and Race/Ethnic-Specific Childhood Metabolic Syndrome Risk Score.** ROC curves and area under the curve (AUC) for childhood MetS Risk Score for non-Hispanic white, non-Hispanic black and Hispanic adolescent participants of NHANES in identifying **A**) “traditional” MetS (ATP III adaptation, Ford 2007) or elevations in **B**) C-reactive protein (CRP), **C**) uric acid, **D**) fasting insulin, or **E**) one or more elevations among CRP, uric acid or fasting insulin or **F**) two or more elevations in these 3 factors. * Elevated CRP = 4.5 or greater; ** Elevated Uric Acid = 5.5 for females; 7.0 for males; † Elevated Fasting Insulin = 16.

Equations based on the factor coefficients from Model 2 are presented in Table 3, and can be used to calculate what is called a MetS risk score based on the clinically-used components of MetS. For ease of clinical use, these equations are based on the actual values of the variables (except for BMI), and not the standardized measures that went into the factor analysis. ROC analysis of this resulting risk score indicates that is has excellent ability to predict a traditional MetS classification (AUC=0.96; Figure 1). It exhibits a moderate ability to discriminate against elevated surrogate outcomes (hsCRP, uric acid, fasting insulin), with AUC values equal to 0.71, 0.75, and 0.82, respectively (Figure 1). However, it has near excellent ability to predict those individuals with ≥2 elevations among the three surrogates (AUC=0.87). Most importantly, the predictive ability assessed here did not systematically differ by sex or race/ethnicity (AUC values ranged between 0.81 and 0.92 across the six groups; not shown).

**Table 3:** Equations for New Sex and Race/Ethnic-Specific Childhood Metabolic Syndrome Risk Z-Score


Males																	
Non-Hispanic White	=	−4.9310	+	0.2804	* BMI Z-score	=	0.0257	* HDL	+	0.0189	* SBP	+	0.6240	* ln(Tri)	+	0.0140	* Glu
Non-Hispanic Black	=	−4.7544	+	0.2401	* BMI Z-score	=	0.0284	* HDL	+	0.0134	* SBP	+	0.6773	* ln(Tri)	+	0.0179	* Glu
Hispanic	=	−3.2971	+	0.2930	* BMI Z-score	=	0.0315	* HDL	+	0.0109	* SBP	+	0.6137	* ln(Tri)	+	0.0095	* Glu
Females																	
Non-Hispanic White	=	−4.3757	+	0.4849	* BMI Z-score	=	0.0176	* HDL	+	0.0257	* SBP	+	0.3172	* ln(Tri)	+	0.0083	* Glu
Non-Hispanic Black	=	−3.7145	+	0.5136	* BMI Z-score	=	0.0190	* HDL	+	0.0131	* SBP	+	0.4442	* ln(Tri)	+	0.0108	* Glu
Hispanic	=	−4.7637	+	0.3520	* BMI Z-score	=	0.0263	* HDL	+	0.0152	* SBP	+	0.6910	* ln(Tri)	+	0.0133	* Glu

**Figure 2:**
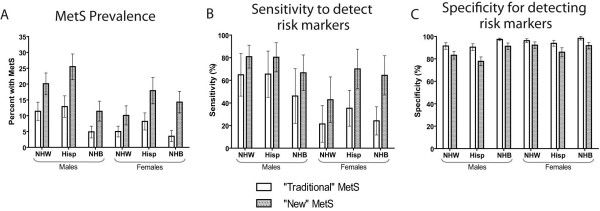
**Comparison of “Traditional” vs. “New” MetS. A**: Prevalence of MetS among adolescents from NHANES 1999–2010 based on a common pediatric adaptation of ATP-III MetS as compared to “New” MetS based on cutoff of new metabolic syndrome risk Z-score = 0.75. **B** and **C**: Sensitivity and specificity ATP-III MetS and “New” MetS in predicting adolescents with two or more elevations of MetS-associated risk markers (hsCRP, uric acid, and fasting insulin). NHW = Non-Hispanic White; NHB = Non-Hispanic Black; Hisp = Hispanic.

From this ROC analysis of ≥2 predictors, a reasonable cut-off of this new continuous score appeared to be around 0.75, if one wished to maintain a traditional MetS binary classification (overall sensitivity=66%; specificity=87%). While using a cutoff is not necessarily advocated here, defining adolescents with a score >0.75 as having “MetS” allowed for a direct comparison of sensitivity values with the traditional MetS criteria in predicting the most severe adolescents, those with ≥2 elevations among hsCRP, uric acid and insulin. When using 0.75, the percent with this new “MetS” is increased considerably compared to the traditional MetS classification across all groups (Figure 2A). While racial/ethnic differences in prevalences remain when using this particular cutoff, particularly for males, the racial/ethnic differences in the ability of a MetS diagnosis to be sensitive and specific to elevated surrogate levels has diminished considerably. As seen elsewhere [14,29,30], the traditional MetS classification has an overall poor sensitivity, and this sensitivity varies by sex and race/ethnicity (Figure 2B). This traditional classification is particularly insensitive for non-Hispanic-black adolescents. However, a new MetS classification using a cutoff of 0.75 is more sensitive, both overall and across sex and race/ethnicity, with no respective clinically meaningful decrease in specificity (Figure 2C). 

## Discussion

The present analysis reveals racial/ethnic and sex differences among adolescents in loading weights to MetS that result in improvements in the ability of MetS to predict elevations in MetS-associated risk markers. While the value of a diagnosis of MetS has been questioned as being more valuable than the sum of its parts [46], the concept of MetS remains a frequently-used research tool that has validity in being able to predict future occurrence of T2DM in children [8] and CVD in adults [17]. Most currently-employed criteria for diagnosing MetS in children and adolescents utilize somewhat arbitrarily-determined cut-off values for individual components (Table 1) that appear to have racial/ethnic biases [25,31] while other sets of criteria have used a sum of z-scores, which does not account for the strong correlations across the components and does not account for possible differential influences of individual components on the overall score [33]. Our current approach offers multiple improvements on these prior approaches by using a confirmatory factor analysis, allowing for sex and racial/ethnic differences in the weighting of individual components to the risk score. Restated, this allows for the possibility that MetS manifests itself differently between sex and racial/ethnic groups in a way that may affect our ability to identify MetS-associated risks. The sex- and race/ethnicity-specific differences result in unique equations to calculate the risk score based on sex and racial/ethnic group. Once this score has been more fully validated it could be placed on an internet web site or smart phone application to assist in clinical use, alerting clinicians and patients regarding an adolescent’s risk score—along with any risk implications specific to that individual’s sex and racial/ethnic group.

In evaluating race/ethnicity-specific differences in this score, we noted in particular variations in contributions of lipid measures by sex and race/ethnicity to the single MetS factor. This is perhaps not surprising, given that non-Hispanic-black adolescents have lower levels of triglycerides and higher levels of HDL at baseline and although they exhibit worsening dyslipidemia with insulin resistance, they are less likely to exhibit gross abnormalities in lipids when using population-based cut-off values [25,27]. This results in a lower prevalence of MetS among non-Hispanic-black males using traditional criteria and a poorer sensitivity for MetS to detect elevations in surrogates of MetS-related processes [14,29,30]. In the current analysis (Table 3), we noted that as compared to the factor loadings for non-Hispanic-white males, non-Hispanic-blacks had a higher loading of HDL (0.51 non-Hispanic whites vs. 0.66 non-Hispanic blacks) but a lower loading of triglycerides (0.62 vs. 0.50). This would suggest that lower levels of HDL may be even more indicative of worsening MetS severity in non-Hispanic-black males compared to non-Hispanic whites while higher levels of triglycerides may not be as strong an indicator in non-Hispanic-black males. Hispanic males exhibited high loading factors of both HDL and triglycerides, potentially suggesting that worsening levels of both components are important indicators of increasing degree of MetS severity. Non-Hispanic-white males had high loadings for SBP (0.50) compared to non-Hispanic blacks (0.33) and Hispanics (0.30). This is interesting given that while elevated SBP is more common in non-Hispanic blacks and less common in Hispanics compared to non-Hispanic whites [14], SBP appears to have a greater relative importance to MetS in non-Hispanic whites compared to the other groups.

Non-Hispanic-white females exhibited lower factor loadings of HDL and triglycerides relative to either of the other racial/ethnic groups, while Hispanic females exhibited the highest loadings for HDL and triglycerides. These data again suggest that changes in these lipid values are more likely to indicate worsening of underlying MetS among Hispanics. Overall, the poor model fit indices for non-Hispanic-white females in particular, may indicate that a one-factor model of MetS may not be appropriate for this group.

It was notable that fasting glucose had a low factor loading for all sex and racial/ethnic groups. While others have included insulin in some manner as a measure in factor analyses [5,33], we chose to use glucose because the lack of standardized assays for insulin would impede use of the risk score for clinical purposes. It has been noted previously that glucose is maintained in a relatively narrow range among obese children [39], and this range was narrowed further in our analysis by excluding diabetic individuals (glucose>125 mg/dL). We ultimately elected to retain glucose in the score because of the near universality of its inclusion in prior MetS criteria [3,7-9,21-24] and its common use in screening for undiagnosed diabetes [47].

Most telling of the accuracy of this new score would be in its ability to predict future disease risk. The optimal testing for such risk would require long-term data including childhood factors and adult disease outcomes. Lacking these, such a score could also be used to assess the quantity of markers associated with processes associated with MetS-related risk, including adiponectin (which appears to be in the causative pathway of insulin resistance [20,48]) or markers of atherogenic dyslipidemia, including ApoB, small, dense LDL particles [49]. In the present analysis, we instead assessed the accuracy of the new score for its ability to identify individuals with elevations in clinical measures that are part of processes related to MetS. These measures were serum levels of fasting insulin (as an assessment of insulin resistance [13]), hsCRP (as an assessment of underlying inflammation [10]) and uric acid (as an assessment of oxidative stress [11,12]). Comparing the score’s performance in predicting these elevations to the more traditional MetS diagnosis required using a cutoff value for the MetS risk score itself—even though one of the benefits of such a score is its lack of binary nature. We chose a cutoff of a z-score of 0.75 based on the ROC curve for the score to identify individuals with traditional MetS. Using this cutoff, there was a higher prevalence of adolescents with MetS in each sex/racial/ethnic group and in particular among non-Hispanic-black males and females (Figure 2A). While traditional MetS criteria (ATP-III based) performed poorly in predicting elevations in these measures (sensitivity 21-65%), the new risk score (cut-off of 0.75) performed significantly better (sensitivity 43-81%) without clinically meaningful differences in specificity (90-98% for traditional score vs. 78-92%). This cut-off, if used, could help to identify a larger number of at-risk children and adolescents that currently-used MetS criteria—particularly among non-Hispanic-black individuals.

It is important to note that our aim was not to question the existence of the metabolic syndrome in general, nor study in an exploratory fashion the precise number of factors. We operated under the assumption that one “MetS” factor exists in the pediatric population, and under the assumption, we assessed whether the components contribute to that factor differentially by sex and race/ethnicity. Along those lines, we focused only on traditional MetS components that are common to almost all existing MetS criteria based on cutoffs of these components in order to ensure a clinically accessible risk score that results from the analysis. The limited number of components that comprise traditional MetS criteria did not allow for examination of more than one factor. Thus, our examination here was to examine the one-factor model of MetS in adolescents that would thus allow for a continuous representation of traditionally-defined MetS while simultaneously allowing for sex and race/ethnic differences within this one-factor model. Our comparison of the predictive ability of this new score to the traditional MetS diagnosis required the use of a cutoff value. However, a clear benefit of this score is its potential to identify elevated risk in an individual who has a high score but would not be classified as having MetS using traditional criteria based on having elevations in components of MetS that did not exceed population-based or perhaps even arbitrary thresholds (see Additional file 1: Table S1 for examples). Importantly, this score could also be used to assess degree of improvement during lifestyle modification treatment for weight loss.

This study had several weaknesses. We utilized NHANES data which, although powerful, are cross-sectional. Future use of this score will need to utilize longitudinal databases that include information regarding long-term disease outcomes. We used BMI z-score as an assessment of obesity. While this has been done for prior MetS criteria [8,39], it is generally recognized that markers of visceral obesity (such as WC) are more strongly associated with MetS risk than BMI [9]. Nevertheless, BMI is known to be highly associated with MetS risk [8,42] and has been noted in prior factor analyses of MetS [1,33]. In addition, our MetS risk score that was developed using BMI z-score had near-perfect ability (ROC AUC=0.96) to discriminate against an ATP-III-based classification that utilized WC percentile cut-offs, indicating use of BMI in this instance is sufficient in the creation of a continuous representation of the traditional MetS diagnosis.

## Conclusions

In summary, using confirmatory factor analysis, we have demonstrated significant sex- and racial/ethnic differences in factor loading of MetS components that has resulted in a novel sex- and race/ethnicity-specific MetS risk score. This continuous score demonstrates strong predictive ability to detect MetS-associated processes while being less prone to racial/ethnic differences than traditional pediatric MetS criteria. Future research is needed to ascertain the ability of this score to identify individuals at risk for long-term CVD and T2DM, as well as its ability to monitor MetS in the setting of lifestyle modification for obesity treatment.

## Abbreviations

AIC: Akaike’s information criteria; AUC: Area under the curve; ATP-III: Adult treatment panel III; BMI: Body mass index; BP: Blood pressure; CVD: Cardiovascular disease; CFA: Confirmatory factor analysis; DBP: Diastolic blood pressure; GFI: Goodness of fit index; HDL: High-density lipoprotein cholesterol; hsCRP: High-sensitivity C-reactive protein; MetS: Metabolic syndrome; NFI: Bentler-bonett normed fit index; NHANES: National health and nutrition examination survey; RMSEA: Root mean square error of approximation; SBP: Systolic blood pressure; SRMR: Standardized root mean square residual; T2DM: Type 2 diabetes mellitus; WC: Waist circumference.

## Competing interests

The authors have no conflicts of interest to disclose.

## Authors’ contributions

MJG helped in the conception and design of the study, acquired the data, analyzed the data and interpreted the results, and wrote the initial draft of the manuscript and contributed in the critical revision process. CLI helped analyzed the data and interpret the results, and critically reviewed and revised the manuscript. SSS helped conceive the study, assisted in the interpretation of the results, and reviewed and revised the manuscript. MDD helped in the conception and design of the study, assisted in the interpretation of the results, and wrote the initial draft of the manuscript and contributed in the critical revision process. All authors read and approved the final manuscript.

## Supplementary Material

Additional file 1 **Table S1.**Proposed Criteria for the Diagnosis of the Metabolic Syndrome in Children. Click here for file
