# Supplementary material for: A confirmatory factor analysis of the metabolic syndrome in adolescents: an examination of sex and racial/ethnic differences
Source: Cardiovasc Diabetol. 2012 Oct 13;11:128. doi: 10.1186/1475-2840-11-128 (PMC3489601; doi:10.1186/1475-2840-11-128)
Supplement: Additional file 1 — Table S1.Proposed Criteria for the Diagnosis of the Metabolic Syndrome in Children. [file 1475-2840-11-128-S1.doc]

Supplementary Table 1: Proposed Criteria for the Diagnosis of the Metabolic Syndrome in Children

|  | Risk factors | Obesity | Hypertension | Hypertriglyceridemia  (mg/dL) | Low HDL  (mg/dL) | Elevated fasting glucose (mg/dL) |
| --- | --- | --- | --- | --- | --- | --- |
|  |  |  |  |  |  |  |
| Ford (2007) | >3 | WC >90% | SBP *or* DBP >90% for age, sex, height | TG >110 mg/dL | HDL ≤ 40 | >100 |
|  |  |  |  |  |  |  |
| IDF  (Zimmet 2007) | >2* | 6-10 y.o.: WC >90% | >130/85 mmHg | TG >150 mg/dL | HDL < 40 | >100 |
|  | >2* | 10-16 y.o.: WC >90% | >130/85 mmHg | TG >150 mg/dL | HDL:  Males <40      Females < 50 | >100 |
|  |  |  |  |  |  |  |
| Morrison (2008) | >2 | BMI >90^th^% | SBP *or* DBP >90% for age, sex, height | TG >110 mg/dL | HDL ≤ 40 | >110 |

 * Elevated WC is a prerequisite and is not counted toward the number of components needed for diagnosis.
